# The Role of Akt in Acquired Cetuximab Resistant Head and Neck Squamous Cell Carcinoma: An *In Vitro* Study on a Novel Combination Strategy

**DOI:** 10.3389/fonc.2021.697967

**Published:** 2021-09-10

**Authors:** Hannah Zaryouh, Ines De Pauw, Hasan Baysal, Patrick Pauwels, Marc Peeters, Jan Baptist Vermorken, Filip Lardon, An Wouters

**Affiliations:** ^1^Center for Oncological Research (CORE), Integrated Personalized & Precision Oncology Network (IPPON), University of Antwerp, Antwerp, Belgium; ^2^Department of Pathology, Antwerp University Hospital, Antwerp, Belgium; ^3^Department of Medical Oncology, Antwerp University Hospital, Antwerp, Belgium

**Keywords:** PI3K/Akt pathway, cetuximab, EGFR, HNSCC, Akt inhibitor, resistance

## Abstract

The epidermal growth factor receptor (EGFR) is a therapeutic target in head and neck squamous cell carcinoma (HNSCC). Resistance to EGFR-targeted therapies, such as cetuximab, poses a challenging problem. This study aims to characterize acquired cetuximab resistance mechanisms in HNSCC cell lines by protein phosphorylation profiling. Through this, promising combination treatments can be identified to possibly overcome acquired cetuximab resistance in HNSCC. Protein phosphorylation profiling showed increased phosphorylation of Akt1/2/3 after cetuximab treatment in acquired cetuximab resistant cells compared to cetuximab sensitive cells, which was confirmed by western blotting. Based on this protein phosphorylation profile, a novel combination treatment with cetuximab and the Akt1/2/3 inhibitor MK2206 was designed. Synergy between cetuximab and MK2206 was observed in two cetuximab sensitive HNSCC cell lines and one acquired cetuximab resistant variant in simultaneous treatment schedules. In conclusion, this study demonstrates that increased Akt1/2/3 phosphorylation seems to be characteristic for acquired cetuximab resistance in HNSCC cell lines. Our results also show an additive to synergistic interaction between cetuximab and MK2206 in simultaneous treatment schedules. These data support the hypothesis that the combination of cetuximab with PI3K/Akt pathway inhibition might be a promising novel therapeutic strategy to overcome acquired cetuximab resistance in HNSCC patients.

## Introduction

Head and neck squamous cell carcinoma (HNSCC) is the sixth most common cancer type worldwide and remains one of the most challenging malignancies to treat (1, 2). Through our increasing knowledge regarding the molecular biology of HNSCC, several therapeutic strategies have been developed. The introduction of targeted therapies that inhibit oncogenic signaling pathways, as well as the development of immunotherapies that activate a patient’s immune system are now at the forefront of personalized medicine in cancer treatment.

Over the past decades, research has revealed that the epidermal growth factor receptor (EGFR, HER1) plays an integral role in the tumorigenesis of HNSCC. Increased or sustained activation of the EGFR signaling pathway can induce malignant transformation through sustained signaling for cell proliferation, anti-apoptotic signaling, angiogenesis and metastasis (3). Furthermore, EGFR is highly expressed in a wide range of malignancies, including HNSCC. As a result, EGFR is considered as a compelling drug target (3, 4).

In 2006, the anti-EGFR monoclonal antibody (mAb) cetuximab, was approved by the Food and Drug Administration (FDA) in combination with radiotherapy for locoregionally advanced HNSCC (median overall survival (OS) of 4.1 years versus 2.4 years with radiotherapy alone) and in combination with platinum-based therapy and infusional 5-fluorouracil (EXTREME regimen) for the first-line treatment of patients with recurrent/metastatic (R/M) HNSCC (median OS 10.1 months versus 7.4 months with chemotherapy alone) (5–7). To date, the therapeutic landscape of HNSCC is changing as the anti-programmed death-1 (PD-1) immune checkpoint inhibitor pembrolizumab has been FDA-approved in June 2019 as a first-line treatment of R/M HNSCC. The Keynote-048 study demonstrated that pembrolizumab with platinum and 5-fluorouracil significantly improved median OS versus the EXTREME regimen with cetuximab in the total population of HNSCC patients (13.0 months versus 10.7 months) (8). In particular, patients expressing programmed death ligand-1 (PD-L1) (85% of HNSCC patients has combined positive score ≥ 1) show an increased benefit from treatment with pembrolizumab plus platinum and 5-fluorouracil (median OS 13.6 months versus 10.4 months). However, response rates to this treatment regimen are 36% and thus still limited and comparable to the EXTREME regimen with cetuximab (8, 9). Hence, to date, management of R/M HNSCC relies on combination treatment involving platinum, 5-fluorouracil and the addition of pembrolizumab or cetuximab. However, as mentioned, only a fraction of HNSCC patients respond to these treatment regimens.

The reason for resistance to pembrolizumab treatment in HNSCC remains largely unknown. In this regard, several biomarkers, such as PD-L1 expression, immune infiltration, tumor mutational burden and immune-gene expression profiling, have been explored for their predictive potential. However, none of them could be validated in HNSCC so far (10). Concerning cetuximab, this resistance is partly attributable to lack and/or loss of sensitivity of tumor cells to EGFR inhibition, which develops during treatment and compromises the therapeutic outcome. If resistance to cetuximab therapy is present at baseline, this is defined as intrinsic (primary) resistance and can be explained by resistance-conferring factors pre-existing in the bulk of tumor cells. Moreover, nearly all patients whose tumors initially respond inevitably become acquired (secondary) resistant. Acquired resistance refers to disease progression during ongoing treatment that was initially effective (11). In these scenarios, targeting EGFR alone may not be efficacious and requires the addition of a supplementary targeting agent to maximize the therapeutic response.

Therefore, the present study aims to investigate a novel combination strategy to overcome acquired cetuximab resistance. We hypothesized that acquired cetuximab resistance in HNSCC may arise from the activation of compensatory signaling pathways following cetuximab treatment, which are able to reverse the inhibitory effects of cetuximab through phosphorylation of key proteins, thereby promoting cell survival. Pharmacological inhibition of these phosphorylated proteins might be essential to overcome acquired cetuximab resistance in HNSCC. Therefore, we first characterized the protein phosphorylation profile of two cetuximab sensitive (Cet_Sens_) HNSCC cell lines and their acquired cetuximab resistant (Acq_Res_) variants. Based on this protein phosphorylation profile, a novel combination treatment was designed to overcome acquired therapy resistance.

## Material and Methods

### Cell Culture

HPV-negative human HNSCC cell lines SC263 and SCC22b were kindly provided by Prof. Dr. Sandra Nuyts (University Hospital Leuven, Leuven, Belgium) and Prof. Dr. Olivier De Wever (Laboratory of Experimental Cancer Research, Ghent University Hospital, Ghent, Belgium). These HNSCC cell lines were previously identified as Cet_Sens_ following cetuximab treatment for 168h using the colorimetric sulforhodamine B (SRB) assay. Isogenic Acq_Res_ HNSCC cell lines (SC263-R and SCC22b-R) were previously generated by chronically exposing these initially Cet_Sens_ cell lines (parental) to cetuximab, starting with the IC_50_ concentration of cetuximab (12–14). In parallel, control cell lines (SC263-S and SCC22b-S) were established by exposure to the vehicle control (PBS). After 10 dose doublings, dose-response studies demonstrated that cetuximab-exposed cells developed resistance towards cetuximab (12–14). All cell lines were cultured in DMEM, supplemented with 10% fetal bovine serum, 1% penicillin/streptomycin and 1% L-glutamine (Life Technologies, Merelbeke, Belgium). Resistant cell lines were exposed to the highest doubling dose of cetuximab every four weeks. Cells were grown as monolayers and maintained in exponential growth in 5% CO_2_/95% air in a humidified incubator at 37°C. All cell lines were confirmed free of mycoplasma infection through regular testing (MycoAlert Mycoplasma Detection Kit, Lonza, Verviers, Belgium). The identity of each cell line was validated through short tandem repeat profiling (Centre of Medical Genetics, University of Antwerp, Antwerp, Belgium).

### Human Protein Phosphorylation Antibody Array

Human phospho-kinase antibody array kit (ARY003B, Proteome Profiler, R&D Systems, Minneapolis, MN, USA) was used to determine the relative levels of protein phosphorylation in the Cet_Sens_ and Acq_Res_ cell lines after cetuximab treatment. The human phospho-kinase antibody array was performed according to the manufacturer’s protocol. In short, cells were lysed on 6-well plates after treatment with cetuximab (0 and 100 nM, 2 hours, diluted in sterile PBS, Merck, Darmstadt, Germany). Twenty minutes before lysis, epidermal growth factor (EGF, 50 ng/ml, Sigma-Aldrich, Diegem, Belgium) was added to the medium. Protein concentrations were determined using the Pierce BCA protein kit (Thermo Scientific, Erembodegem, Belgium). Next, samples were prepared and 450 μg of proteins were incubated overnight with nitrocellulose membranes which are spotted with 46 capture antibodies in duplicate. The specific target proteins (**Supplementary Table 1**), if present in the sample, bind to these capture antibodies, leading to protein-antibody interactions. These protein-antibody interactions are then visualized using chemiluminescent detection reagents on the Lumi-Imager (Roche Diagnostics, Vilvoorde, Belgium). The antibodies of this kit bind to all isoforms of the target proteins and are therefore not isoform-specific. The signal is proportional to the amount phosphorylation in the bound analyte. Quantification of phosphorylation levels was executed following the manufacturer’s protocol. In short, the integrated optical density of each spot was measured and corrected for background signal using Image J software (15). Only proteins that gave rise to an integrated optical density at least 1.5-fold above background were further used for data processing. Mean integrated optical densities were obtained by averaging the integrated optical density of the duplicates on the array. The fold changes were calculated by dividing the mean integrated optical densities of the treatment and control groups.

### Western Blot

In order to validate findings of the human phospho-kinase antibody array with western blot, cell lysates were three times independently prepared as described above. Twenty micrograms of proteins were separated by SDS-page (10% polyacrylamide gel, 100V, 2h) and western blot was performed (100V, 1h). Blocking of non-specific binding sites was done by incubation of the membrane with Odyssey blocking buffer TBS (Li-Cor, Leusden, The Netherlands) for 1h at room temperature while shaking. Next, primary and secondary antibody incubation and washing was performed using the SNAP id 2.0 protein detection system (Merck Millipore) according to the manufacturer’s instructions. Membranes were incubated with the following antibodies: phospho-Akt (S473) rabbit mAb (1:1000, no. 193H12, Cell Signaling Technology, Leiden, The Netherlands) and total Akt (pan) rabbit mAb (1:1000, no. 11E7, Cell Signaling Technology). Anti-β-actin was used as an internal standard (1:5000, no. A5441, Sigma Aldrich). Goat-anti-rabbit (1:10000, no. 926-32211, Li-Cor) or goat-anti-mouse (1:10000, no. 926-68070, Li-Cor) fluorescently labeled secondary antibodies were used and fluorescent detection was performed using the Odyssey imaging system (Li-Cor). Protein levels were quantified using Image Studio™ Lite, a software program available on the Odyssey imaging system. Phospho-Akt and Akt expression levels were corrected for loading differences based on beta-actin expression. Fold changes were calculated by dividing the mean fluorescent signal of the treatment and control groups.

### *In Silico* Analysis of Akt1, Akt2 and Akt3 Expression

The baseline mRNA expression of Akt1/2/3 in HNSCC patients was examined using the RNA sequencing data from The Cancer Genome Atlas (TCGA) dataset (Provisional, 522 sequenced HNSCC patients). RNASeqV2 from TCGA was processed and normalized using the software package RNA-Seq by Expectation Maximization (RSEM) in order to generate transcripts per million. This dataset was downloaded from cBioportal.

### Sulforhodamine B Assay

Cell survival was assessed using the colorimetric SRB assay, as previously described (16, 17). This assay is used for cell density determination, based on the measurement of cellular protein content, whereby it is not possible to make a distinction between inhibition of proliferation (cytostatic effect) and cell death (cytotoxic effect). Optimal seeding densities for each cell line were determined in order to ensure exponential growth during the whole duration of the assay. Cells were counted with a TC20 Automated Cell Counter (Biorad, Temse, Belgium). After overnight incubation, cells were treated with MK2206 (72h, 0-5 μM, pan-Akt inhibitor, Selleck Chemicals, Houston, USA) in combination with cetuximab (0-50 nM). Hereby, two simultaneous combination schedules were tested:

1. Cetuximab plus MK2206 with total treatment duration of 72h
2. Cetuximab for 168h with MK2206 added during the last 72h of treatment

MK2206 was diluted in DMSO (Merck Millipore SA/NV, Overijse, Belgium) and further dilutions were made in cell culture medium. IC_50_ values (i.e. drug concentration causing 50% growth inhibition) were calculated using Graphpad Prism 9 software. Possible synergism between cetuximab and MK2206 was determined by calculation of the combination index (CI) using the Additive Model as described by others (18–20). CI < 0.800, CI = 1.000 ± 0.200 and CI > 1.200 indicated synergism, additivity and antagonism, respectively.

### Statistical Analysis

We performed all experiments at least three times independently, unless otherwise stated. In cytotoxicity experiments, each condition was tested in triplicate in each of the three experiments. Differences in total and phosphorylated Akt1/2/3, determined with western blot, were statistically analyzed using the non-parametric Kruskal-Wallis test. Significant values were adjusted by the Bonferroni correction for multiple testing. One-way ANOVA with Tukey-Kramer HSD posthoc analysis was used to assess significant differences in Akt1/2/3 mRNA expression of HNSCC patients (RNASeqV2 TCGA data). The influence of cetuximab treatment on the effect of MK2206 alone was evaluated for each cell line using linear mixed models in case of non-independent observations. More specifically, combination treatment was set as fixed effect. A random intercept for experimental replicates was added in order to account for the dependence of observations between experiments. The influence of cetuximab resistance status on the effect of MK2206 alone was also evaluated using linear mixed models. Resistance status was set as fixed effect and a random intercept for cell line and experimental replicates was added to account for the dependence of observations between cell lines and experiments. GraphPad Prism 9 was used for data comparison and artwork. All statistical analyses were performed in JMP Pro 15 and SPSS v25. P-values below 0.050 were considered significant.

## Results

### Acquired Cetuximab Resistant HNSCC Cell Lines Show Increased Phosphorylation of Akt After Cetuximab Treatment

In order to characterize acquired cetuximab resistance, a protein phosphorylation profile was established using the human phospho-kinase antibody array. Hereby, the effect of cetuximab treatment on the phosphorylation of various proteins (**Supplementary Table 1**) was determined in two Cet_Sens_ HNSCC cell lines and their isogenic Acq_Res_ variants. Data analysis of the protein phosphorylation profile revealed a differential response of Cet_Sens_ HNSCC cell lines and Acq_Res_ variants to cetuximab treatment. As this change was most pronounced in substrates involved in the Akt signaling pathway, we focused further on the interpretation of these results in the next paragraph.

**Figure 1:**
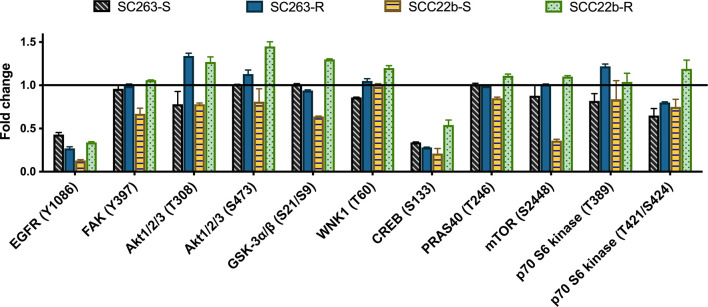
Quantification of the human phospho-kinase array blots of cetuximab sensitive (SC263-S and SCC22b-S) HNSCC cell lines and corresponding acquired cetuximab resistant (SC263-R and SCC22b-R) variants. Graph represents the fold change of selected substrates, i.e. phosphorylated EGFR, Akt1/2/3 and other substrates involved in the Akt pathway, after cetuximab treatment. Protein phosphorylation profiling was conducted with one cell lysate. The fold changes were calculated by the ratio of the mean integrated optical density (duplicates) in treatment versus control groups. Error bars were calculated based on differences between duplicates. Suffix -S: cetuximab sensitive cell line and suffix -R: acquired cetuximab resistant cell line.

The effects of cetuximab treatment on the phosphorylation of EGFR, Akt and other substrates involved in the Akt pathway were quantified in Cet_Sens_ HNSCC cell lines and Acq_Res_ variants (**Figure 1**). Following cetuximab treatment, activating phosphorylation of EGFR was decreased in all HNSCC cell lines, suggesting inhibition of EGFR signaling. Both Cet_Sens_ HNSCC cell lines (i.e. SC263-S and SCC22b-S) demonstrated decreased phosphorylation of Akt1/2/3 at T308 after cetuximab treatment. T308 is phosphorylated by phosphoinositide-dependent kinase-1 (PDK1) in the phosphoinositide 3-kinase (PI3K)/Akt pathway, leading to Akt activation (21). As a result, the PI3K/Akt pathway is inhibited by cetuximab in Cet_Sens_ HNSCC cell lines. In contrast, a considerable increase in Akt1/2/3 phosphorylation at T308 was observed after cetuximab treatment in both Acq_Res_ variants. Furthermore, phosphorylation of Akt1/2/3 at S473, by mammalian target of rapamycin complex 2 (mTORC2) (21), was also enhanced in both Acq_Res_ variants, particularly in SCC22b-R. In addition, cetuximab treatment induced an increased phosphorylation of several downstream substrates of the Akt pathway [e.g. mTOR, p70 S6 kinase, glycogen synthase kinase-3 α/ß (GSK-3α/ß), proline-rich Akt substrate 40kDa (PRAS40) and WNK lysine deficient protein kinase-1 (WNK1)] in Acq_Res_ variants, especially in SCC22b-R. This means that the Akt pathway is still activated in Acq_Res_ cells following cetuximab treatment, leading to anti-apoptotic and pro-proliferative effects.

### Western Blot Analysis Confirms Increased Akt1/2/3 Phosphorylation Following Cetuximab Treatment in Acquired Cetuximab Resistant HNSCC Cell Lines

**Figure 2:**
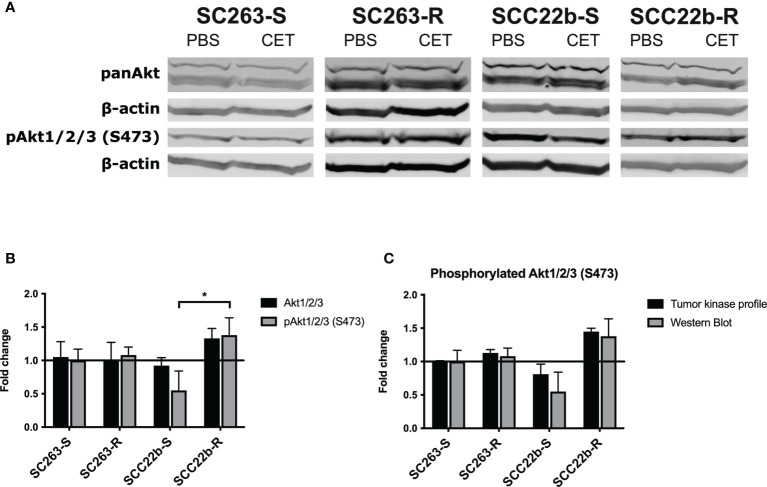
Protein levels of total and phosphorylated Akt1/2/3 in cetuximab sensitive (SC263-S and SCC22b-S) HNSCC cell lines and corresponding acquired cetuximab resistant (SC263-R and SCC22b-R) variants. **(A)** Western blot was used to determine the levels of total and phosphorylated Akt1/2/3 (S473) after cetuximab treatment in cetuximab sensitive and acquired cetuximab resistant HNSCC cell lines. β-actin detection served as loading control. **(B)** Quantification of western blot results for total and phosphorylated Akt1/2/3. Signals were corrected for β-actin and the effect of cetuximab treatment is presented as fold change. Fold changes were calculated by dividing the mean fluorescent signal of the treatment and control groups. The graph represents the average fold change of three independent experiments. **(C)** Fold change of phosphorylated Akt1/2/3 at S473 following cetuximab treatment observed with protein phosphorylation profiling and western blot. CET, cetuximab; pAkt1/2/3, phosphorylated Akt1/2/3; *p-value ≤ 0.050. Suffix -S: cetuximab sensitive cell line and suffix -R: acquired cetuximab resistant cell line.

Results from the protein phosphorylation profiling were first validated using western blot. Protein levels of total Akt1/2/3 and phosphorylated Akt1/2/3 at S473 were determined after cetuximab treatment in Cet_Sens_ HNSCC cell lines and Acq_Res_ variants (**Figures 2A, B**). Regarding the expression of total Akt 1/2/3, no change was observed following cetuximab treatment in the SC263-S and the Acq_Res_ SC263-R variant. Cetuximab treatment resulted in a small decrease in total Akt1/2/3 in SCC22b-S, while it resulted in a considerable increase in the Acq_Res_ variant (i.e. SCC22b-R). There was no statistical difference in the change of expression of total Akt1/2/3 following cetuximab treatment across cell lines (p = 0.130, **Figure 2B**). Cetuximab treatment did not induce any change in the phosphorylation of Akt1/2/3 at S473 in SC263-S, while a clear decrease in phosphorylated Akt1/2/3 was observed in SCC22b-S after cetuximab treatment. Cetuximab treatment induced a very small increase in the phosphorylation of Akt1/2/3 in SC263-R, while a considerable increase was observed in SCC22b-R. These results suggest a compensatory activation of the PI3K/Akt pathway in reaction to cetuximab treatment in Acq_res_ variants. Statistical analysis showed a significant difference in the change of Akt1/2/3 phosphorylation at S473 after cetuximab treatment between SCC22b-S and SCC22b-R (p = 0.023), but not between SC263-S and SC263-R (p = 1.000, **Figure 2B**). Comparison of the results of the protein phosphorylation profiling and western blot showed good similarity between both assays. Hence, the increase in Akt1/2/3 phosphorylation after cetuximab treatment in the Acq_Res_ HNSCC cell lines, detected with protein phosphorylation profiling, was confirmed with western blot (**Figure 2C**).

In conclusion, protein phosphorylation profiling demonstrated increased phosphorylation of Akt1/2/3 after cetuximab treatment in the Acq_Res_ HNSCC cell lines. This increase in phosphorylation was confirmed with western blot and was found significantly different between SCC22b-S and SCC22b-R. These results suggest that the combination of cetuximab with an Akt1/2/3 inhibitor might be a potential novel therapeutic combination strategy to overcome acquired cetuximab resistance in HNSCC cell lines.

### HNSCC Patients Demonstrate Akt Expression

Before investigating the cytotoxic effect of the combination treatment with the EGFR inhibitor cetuximab and the Akt1/2/3 inhibitor MK2206, we determined the expression of Akt isoforms in HNSCC patients using RNA sequencing data from the TCGA dataset (Provisional, RNASeqV2 RSEM, 522 sequenced HNSCC patients). This data demonstrated that there is a significant difference in mRNA expression between Akt1, Akt2 and Akt3 (p < 0.0001, **Supplementary Table 2**) in HNSCC patients (**Supplementary Figure 1**). As substantial Akt1, Akt2 and Akt3 mRNA levels were detected in the majority of tumor samples of HNSCC patients, we can conclude that the target of MK2206 is present in HNSCC patients. Therefore, this drug can play a potential role in the treatment of HNSCC patients.

### Combining Cetuximab With MK2206 Shows Additive to Synergistic Effects in HNSCC Cell Lines

Possible synergistic effects of treatment with cetuximab and MK2206 were investigated in two Cet_Sens_ HNSCC cell lines and their isogenic Acq_Res_ variants. Two simultaneous combination schedules were evaluated. First, cells were treated simultaneously for 72h with 0-5 µM MK2206 and fixed doses of cetuximab. Fixed doses of cetuximab were based on the outcome of previous monotherapy experiments with total treatment duration of 72h (data not shown). As mentioned above, we previously identified Cet_Sens_ cell lines based on the outcomes of experiments with a total treatment time of 168h (14). After 72h of cetuximab treatment, there was only a limited effect on cell survival. Therefore, we chose two high concentrations of cetuximab (i.e. 25 nM and 50 nM) to use in these simultaneous combination experiments with MK2206. Based on results reported in clinical trials, these concentrations are considered clinically relevant in *in vitro* studies (22). Regarding MK2206, based on early phase clinical trials, clinically relevant concentrations with manageable side effects were estimated at ≤1 µM for *in vitro* studies (23).

**Figure 3:**
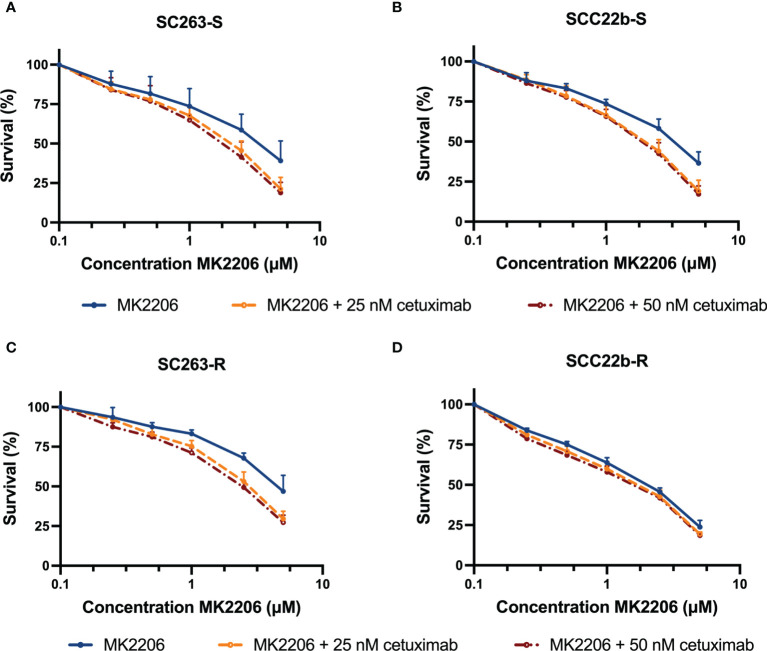
The cytotoxic effect of cetuximab plus MK2206 with a total treatment duration of 72h. Dose-response curves for the cetuximab sensitive HNSCC cell lines SC263-S **(A)** and SCC22b-S **(B)** show a synergistic effect. Regarding the acquired cetuximab resistant variants, dose-response curves indicate a synergistic and additive effect in SC263-R **(C)** and SCC22b-R **(D)**, respectively. Survival curves were corrected for the cytotoxic effect of cetuximab alone. Cells were treated with fixed concentrations of cetuximab, which were chosen based on the outcome of previous monotherapy experiments. Suffix -S: cetuximab sensitive cell line and suffix -R: acquired cetuximab resistant cell line.

The dose-response curves of the Cet_Sens_ HNSCC cell lines and their Acq_Res_ variants are shown in **Figures 3A–D**. A clear concentration-dependent effect of MK2206 after 72 hours of treatment was observed in all HNSCC cell lines. The IC_50_ values for MK2206 monotherapy ranged from 1.752 ± 0.084 µM to 4.764 ± 0.436 µM (**Supplementary Figure 2A** and **Table 1**). Acquired cetuximab resistance had no significant influence on the inhibitory potential of MK2206 (p = 0.954). Compared to MK2206 treatment alone, simultaneous treatment with cetuximab caused a significant decrease in IC_50_ value (p ≤ 0.048) in Cet_Sens_ HNSCC cell lines and the Acq_Res_ variants (**Supplementary Figure 2A** and **Table 1**). Furthermore, the CI ranged from 0.707 to 0.917 (**Table 2**). Synergy between cetuximab and 2.5 µM MK2206 was observed in two Cet_Sens_ HNSCC cell lines and one Acq_Res_ variant (i.e. in SC263-S, SC263-R and SCC22b-S with CI ≤ 0.783, **Supplementary Figure 2B** and **Table 2**) after 72h of simultaneous treatment. Combining MK2206 with higher cetuximab concentrations resulted in an increased synergistic or additive effect.

**Table 1:** IC_50_ and standard errors for HNSCC cell lines after treatment with MK2206 plus cetuximab for 72h.

Cell line	Condition	IC_50_ MK2206 (µM)	P-value
**SC263-S**	MK2206	3.311 ± 0.537	–
	MK2206 + 25 nM cetuximab	1.816 ± 0.134	0.068
	MK2206 + 50 nM cetuximab	1.609 ± 0.154	**0.048**
**SCC22b-S**	MK2206	3.086 ± 0.239	–
	MK2206 + 25 nM cetuximab	1.749 ± 0.120	**0.006**
	MK2206 + 50 nM cetuximab	1.646 ± 0.101	**0.004**
**SC263-R**	MK2206	4.764 ± 0.436	–
	MK2206 + 25 nM cetuximab	2.545 ± 0.135	**0.015**
	MK2206 + 50 nM cetuximab	2.212 ± 0.109	**0.010**
**SCC22b-R**	MK2206	1.752 ± 0.084	–
	MK2206 + 25 nM cetuximab	1.446 ± 0.085	**0.020**
	MK2206 + 50 nM cetuximab	1.327 ± 0.082	**0.006**

P < 0.050, significant difference in IC_50_ compared to MK2206 monotherapy. P < 0.050 are indicated in bold. -, cannot be calculated. Suffix -S: cetuximab sensitive cell line and suffix -R: acquired cetuximab resistant cell line.

**Table 2:** CI for HNSCC cell lines after treatment with cetuximab plus 2.5 µM MK2206 for 72h.

Cell line	Condition	CI
**SC263-S**	2.5 µM MK2206 + 25 nM cetuximab	**0.775**
	2.5 µM MK2206 + 50 nM cetuximab	**0.707**
**SCC22b-S**	2.5 µM MK2206 + 25 nM cetuximab	**0.757**
	2.5 µM MK2206 + 50 nM cetuximab	**0.730**
**SC263-R**	2.5 µM MK2206 + 25 nM cetuximab	**0.783**
	2.5 µM MK2206 + 50 nM cetuximab	**0.728**
**SCC22b-R**	2.5 µM MK2206 + 25 nM cetuximab	0.937
	2.5 µM MK2206 + 50 nM cetuximab	0.917

CI < 0.800, CI = 1.000 ± 0.200, and CI > 1.200 indicate synergism, additivity or antagonism, respectively. CI < 0.800 are indicated in bold. Suffix -S: cetuximab sensitive cell line and suffix -R: acquired cetuximab resistant cell line.

Next, the cytotoxic effect of the second treatment schedule was investigated. Hereby, the cells were treated with fixed doses of cetuximab for 168h and MK2206 (0-2.5 µM) was added during the last 72h of treatment. The goal of this prolonged treatment schedule was to decrease the used drug concentrations. In contrast to the first treatment schedule, Cet_Sens_ HNSCC cell lines were treated with lower concentrations of cetuximab (i.e. 0.5 nM, 1 nM and 5 nM) due to prolonged treatment duration. These fixed concentrations were based on the outcome of previous monotherapy experiments with a total treatment duration of 168h (14).

**Figure 4:**
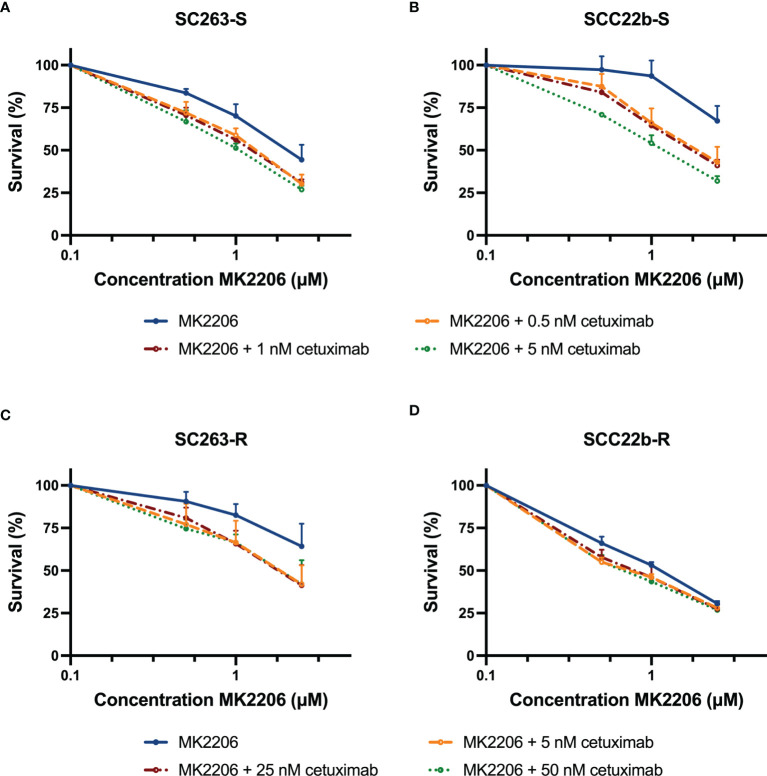
The cytotoxic effect of cetuximab for 168h with MK2206 added during the last 72h of treatment. Dose-response curves for the cetuximab sensitive HNSCC cell lines SC263-S **(A)** and SCC22b-S **(B)** show a synergistic interaction. Regarding the acquired cetuximab resistant variants, dose-response curves indicate a synergistic and additive effect in SC263-R **(C)** and SCC22b-R **(D)**, respectively. Survival curves were corrected for the cytotoxic effect of cetuximab alone. Cells were treated with fixed concentrations of cetuximab, which were based on the outcome of previous monotherapy experiments. Suffix -S: cetuximab sensitive cell line and suffix -R: acquired cetuximab resistant cell line.

**Figures 4A–D** shows the dose-response curves of the Cet_Sens_ HNSCC cell lines and their Acq_Res_ variants. The addition of MK2206 during the last 72h of treatment still resulted in a concentration-dependent cytotoxic effect of this compound. The IC_50_ values for MK2206 ranged from 1.073 ± 0.038 µM to 4.372 ± 1.182 µM (**Supplementary Figures 3A, B** and **Table 3**). Compared to MK2206 treatment alone, simultaneous treatment with cetuximab resulted in a decrease in IC_50_ value (0.033 ≤ p ≤ 0.127). Furthermore, in this treatment regimen, the CI ranged from 0.578 to 0.867 (**Table 4**). Synergy between cetuximab and 1 µM MK2206 was observed in two Cet_Sens_ HNSCC cell lines and one Acq_Res_ variant (i.e. in SC263-S, SC263-R and SCC22b-S with CI ≤ 0.796, **Supplementary Figures 3C, D** and **Table 4**). Combining MK2206 with higher cetuximab concentrations increased the synergistic interaction in Cet_Sens_ cell lines. As this synergistic effect was reached using lower concentrations of both MK2206 and cetuximab, we consider this treatment schedule as the most interesting for further investigation.

**Table 3:** IC_50_ and standard errors for HNSCC cell lines after treatment with cetuximab for 168h with MK2206 added during the last 72h of treatment.

Cell line	Condition	IC_50_ MK2206 (µM)	P-value
**SC263-S**	MK2206	2.067 ± 0.177	–
	MK2206 + 0.5 nM cetuximab	1.253 ± 0.078	0.127
	MK2206 + 1 nM cetuximab	1.200 ± 0.043	0.102
	MK2206 + 5 nM cetuximab	1.002 ± 0.049	0.051
**SCC22b-S**	MK2206	3.541 ± 0.540	–
	MK2206 + 0.5 nM cetuximab	1.919 ± 0.197	0.112
	MK2206 + 1 nM cetuximab	1.787 ± 0.071	0.082
	MK2206 + 5 nM cetuximab	1.187 ± 0.043	**0.027**
**SC263-R**	MK2206	4.372 ± 1.182	–
	MK2206 + 5 nM cetuximab	1.851 ± 0.315	**0.045**
	MK2206 + 25 nM cetuximab	1.815 ± 0.201	0.054
	MK2206 + 50 nM cetuximab	1.51 ± 0.264	0.090
**SCC22b-R**	MK2206	1.073 ± 0.038	–
	MK2206 + 5 nM cetuximab	0.710 ± 0.034	0.052
	MK2206 + 25 nM cetuximab	0.769 ± 0.061	0.102
	MK2206 + 50 nM cetuximab	0.670 ± 0.033	**0.033**

P < 0.050, significant difference in IC_50_ compared to MK2206 monotherapy. P < 0.050 are indicated in bold. -, cannot be calculated. Suffix -S: cetuximab sensitive cell line and suffix -R: acquired cetuximab resistant cell line.

**Table 4:** CI for HNSCC cell lines after treatment with cetuximab for 168h with 1 µM MK2206 added during the last 72h of treatment.

Cell line	Condition	CI
**SC263-S**	1 µM MK2206 + 0.5 nM cetuximab	0.838
	1 µM MK2206 + 1 nM cetuximab	0.801
	1 µM MK2206 + 5 nM cetuximab	**0.732**
**SCC22b-S**	1 µM MK2206 + 0.5 nM cetuximab	**0.705**
	1 µM MK2206 + 1 nM cetuximab	**0.688**
	1 µM MK2206 + 5 nM cetuximab	**0.578**
**SC263-R**	1 µM MK2206 + 5 nM cetuximab	0.805
	1 µM MK2206 + 25 nM cetuximab	**0.796**
	1 µM MK2206 + 50 nM cetuximab	0.806
**SCC22b-R**	1 µM MK2206 + 5 nM cetuximab	0.867
	1 µM MK2206 + 25 nM cetuximab	0.865
	1 µM MK2206 + 50 nM cetuximab	0.818

CI < 0.800, CI = 1.000 ± 0.200, and CI > 1.200 indicate synergism, additivity or antagonism, respectively. CI < 0.800 are indicated in bold. Suffix -S: cetuximab sensitive cell line and suffix -R: acquired cetuximab resistant cell line.

## Discussion

Targeted therapies are key for the personalized treatment of cancer patients (3). Although treatment with the EGFR-inhibitor cetuximab improves OS in HNSCC patients, therapeutic resistance poses a challenging problem and limits the success of effective anti-EGFR cancer therapies in the clinic (24). Therefore, it is of utmost importance to rationally develop novel combination strategies to overcome this therapy resistance.

Increased or sustained stimulation of EGFR mediates the activation of various signal transduction pathways. Proteins involved in these signaling pathways are potential contributors to the development of acquired resistance to drugs inhibiting EGFR signaling (25). In order to identify the signaling pathways characteristic for acquired cetuximab resistance, protein phosphorylation profiling was performed in HNSCC cell lines. This technique has already been successfully applied in other studies to identify drug resistance mechanisms (26–28). In the present study, the effect of cetuximab treatment on the phosphorylation of several proteins was determined in two Cet_Sens_ HNSCC cell lines and their isogenic Acq_Res_ variants. Based on this protein phosphorylation profiling, novel rationally designed combination strategies were investigated to overcome drug resistance.

Protein phosphorylation profiling showed a differential response of Cet_Sens_ HNSCC cell lines and their Acq_Res_ variants to EGFR inhibition by cetuximab. This profile strongly suggested that increased phosphorylation of Akt1/2/3 following cetuximab treatment is characteristic for Acq_Res_ HNSCC cell lines. In general, Cet_Sens_ HNSCC cell lines demonstrated decreased phosphorylation of EGFR (Y1086), Akt1/2/3 (T308 and S473) and downstream substrates after cetuximab treatment. Although EGFR phosphorylation was decreased, Akt1/2/3 was still phosphorylated and activated following cetuximab treatment in Acq_Res_ HNSCC cell lines. This was supported by the observed increase in phosphorylation of several downstream substrates of Akt, such as mTOR, p70S6 kinase, GSK-3α/β, PRAS40 and WNK1. Thus, in Acq_Res_ HNSCC cell lines, the Akt pathway was still able to exerts its anti-apoptotic and pro-proliferative effects under cetuximab treatment, possibly leading to therapy resistance. Importantly, it is worth mentioning that the number of cell lines used in this study is a limiting factor. Therefore, our data needs to be validated in additional isogenic HNSCC cell lines (and ideally in HNSCC patient samples) in order to strengthen the results. Although further confirmation in HNSCC cell lines and patient samples is still needed, based on our results, Akt represents a potential target to improve outcome of cetuximab-based treatment in HNSCC patients.

The PI3K/Akt pathway has been shown to regulate various normal biological processes, such as cellular survival, migration, proliferation, differentiation, angiogenesis, protein synthesis and glucose metabolism. Activation of Akt can be initiated by several events, mainly through a receptor-ligand interaction on the cell membrane. This receptor activation results in activation of PI3K, which phosphorylates phosphatidylinositol 3,4-biphosphate (PIP_2_) to generate phosphatidylinositol 3,4,5-triphosphate (PIP_3_). The binding of PIP_3_ to Akt locates Akt to the plasma membrane and allows its phosphorylation and activation by PDK1. Akt can also be phosphorylated by other substrates and in response to cellular stress, such as ischemia, hypoxia and oxidative stress. The tumor suppressor phosphatase and tensin homolog (PTEN) catalyzes the dephosphorylation of PIP_3_ and is the major negative regulator of Akt signaling. Activated Akt exerts its effects by phosphorylating various downstream substrates, all resulting in anti-apoptotic or pro-proliferative effects (29–32). However, the pathway is also associated with a number of oncogenic processes. Indeed, the PI3K/Akt pathway is one of the most frequently dysregulated signaling pathways in cancer, including HNSCC (33, 34).

The present study demonstrates that increased Akt activation (not isoform-specific) is characteristic for acquired cetuximab resistance in HNSCC cell lines. Importantly, this observation needs to be validated in a cohort of HNSCC patient samples in order to elucidate its clinical value. Nevertheless, previous research has already suggested that enhanced Akt activation can play a role in resistance to cetuximab, not only in HNSCC but also in colorectal cancer and non-small cell lung cancer (35–39). Increased activation of the Akt signaling pathway has been associated with genetic alterations in the *PIK3CA* gene (40). For instance, Rebucci et al. have demonstrated that treatment of cetuximab resistant HNSCC cells with cetuximab did not result in the decreased levels of phosphorylated Akt that were seen in cetuximab sensitive HNSCC cells. A mutation in exon 20 of the *PIK3CA* gene, that encodes for the catalytic p110 α subunit of PI3K, was responsible for this persistent Akt activation (36). According to the TCGA dataset, respectively 18.4% and 20.8% of HNSCC patients demonstrate *PIK3CA* mutations or amplification (41, 42). In addition, loss of the tumor suppressor *PTEN* can also lead to persistent activation of the PI3K/Akt pathway (43, 44). According to TCGA, deep and shallow deletions in the *PTEN* gene occur in 3.4% and 24.0% of HNSCC patients, respectively (41, 42). Based on these findings, the genetic background of the HNSCC cell lines used in this study was determined using whole-exome sequencing (data not shown). Both Cet_Sens_ HNSCC cell lines and their Acq_Res_ variants display no mutations, deletions and/or insertions in the *PI3KCA* and *PTEN* genes. As such, the persistent Akt activation in Acq_Res_ HNSCC cell lines following cetuximab treatment cannot be explained by the genetic background of the cell lines. However, genetic alterations in *PIK3CA* and *PTEN*, possibly leading to persistent Akt activation, are present in a significant number of HNSCC patients in the TCGA cohort. Interestingly, both Cet_Sens_ HNSCC cell lines and their Acq_Res_ variants have *TP53* gene mutations that are predicted to have a deleterious effect on protein function. Functional p53 inhibits the PI3K/Akt pathway by regulating the transcription of four genes, which all have an inhibitory effect on Akt and mTOR (45, 46). As a result, mutant p53 can cause sustained activation of the PI3K/Akt pathway. However, as not only Acq_Res_ cell lines, but also Cet_Sens_ cell lines display mutations in *TP53*, the underlying mechanism behind the observed increased phosphorylation of Akt after cetuximab treatment in Acq_Res_ HNSCC cell lines remains unclear. To define the exact role of genetic alterations in the PI3K/Akt pathway, regarding response to cetuximab, more in-depth studies are needed with HNSCC patient samples.

As we found that increased Akt1/2/3 phosphorylation is characteristic for Acq_Res_ HNSCC cell lines and Akt1, Akt2 and Akt3 are expressed in HNSCC patients, we hypothesized that the combination of cetuximab with an Akt inhibitor might be a potential novel therapeutic strategy to overcome acquired cetuximab resistance. To date, Akt inhibitors are not yet included in clinical practice (32). A phase II study with the pan-Akt inhibitor MK2206 in R/M HNSCC patients showed promising results (i.e. partial responses), but was not moved to phase III so far (NCT01349933). This might be due to the fact that pan-Akt inhibitors targeting all isoforms of Akt have shown to enhance the invasiveness of cancer cells in some cases. In this regard, Brolih et al. demonstrated that Akt1 inhibition leads to a more invasive phenotype in HNSCC tumors that primarily express Akt1 (47). Consistently, the latter was also observed in several studies for breast cancer (48–51). This suggests that the expression of specific Akt isoforms can influence the outcome of pharmacological Akt inhibition. Therefore, Akt isoform analysis may be necessary in order to predict the outcome of pan-Akt inhibitors (47). Alternatively, the use of isoform-selective Akt inhibitors, which have recently been developed (52), may also offer a solution to overcome the potential limitations of the pan-Akt inhibitor MK2206. On the other hand, it has been suggested that future studies should explore mechanism-based combination strategies with chemotherapy or other molecular targeted agents (53).

In the present study, our results provide a rationale to combine cetuximab with MK2206 to overcome acquired cetuximab resistance in HNSCC. To test our hypothesis, we examined the effects of treatment with cetuximab and the Akt inhibitor MK2206 in Cet_Sens_ HNSCC cell lines and Acq_Res_ variants. Hereby, two simultaneous combination schedules were tested. Synergy between cetuximab and MK2206 was observed in two Cet_Sens_ HNSCC cell lines and one Acq_Res_ variant in both simultaneous treatment schedules. An additive effect was observed in the Acq_Res_ SCC22b-R HNSCC cell line. Interestingly, MK2206 monotherapy demonstrated already a large cytotoxic effect in the SCC22b-R cell line compared to the other three cell lines used in this study. A possible explanation is that the SCC22b-R cell line demonstrated the largest increase in phosphorylation of Akt1/2/3 in the protein phosphorylation profiling analysis. As a result, the increased cytotoxic effect of MK2206 treatment alone might explain the observed additive interaction with cetuximab. Additional research is required to further elucidate the molecular mechanisms underlying the additive to synergistic effect between cetuximab and MK2206. Overall, this study demonstrated the potential of Akt inhibition in combination treatments to overcome acquired cetuximab resistance in HNSCC.

Besides the rational design of novel combination strategies, characterization of cetuximab resistance mechanisms can also lead to the identification of predictive biomarkers. To date, no definitive biomarkers have been identified to predict the efficacy of EGFR-targeting agents in patients with HNSCC (25, 54). We found in literature that phosphorylation of Akt at S473 serves as an independent prognostic marker for radiosensitivity in advanced HNSCC and that inhibition of Akt phosphorylation with pharmacological compounds might circumvent resistance to radiotherapy (55). Moreover, it has already been reported that phosphorylated Akt might be a potential predictive biomarker for EGFR-targeted therapies. For instance, lower phosphorylated Akt was observed in cetuximab sensitive HNSCC tumors in cell line xenograft models (56). Furthermore, Lyuo et al. analyzed a cohort with 50 oral squamous cell carcinoma patients who received cetuximab-based induction chemotherapy and found that lower expression of phosphorylated Akt was associated with better disease-free survival (57). In addition, the ECOG2303 phase II trial showed that biomarker signatures consistent with activation of the PI3K/Akt pathway are associated with inferior outcomes to cetuximab-containing chemoradiotherapy regimen (37). These results encourage additional research to precisely define the role of the PI3K/Akt pathway as predictive biomarker for EGFR-targeting agents in HNSCC.

## Conclusion

In conclusion, protein phosphorylation analysis demonstrated that increased Akt1/2/3 phosphorylation is characteristic for acquired cetuximab resistance in HNSCC cell lines. Furthermore, we observed an additive to synergistic interaction between the EGFR inhibitor cetuximab and the pan-Akt inhibitor MK2206 in cetuximab sensitive and acquired cetuximab resistant HNSCC cell lines. Overall, these data support the hypothesis that downstream effectors of the PI3K/Akt pathway serve as promising drug targets in the search for novel therapeutic combination strategies that are able to overcome resistance to anti-EGFR treatment in HNSCC patients.

## Data Availability Statement

All data generated or analysed during this study are available from the corresponding author on reasonable request.

## Ethics Statement

Ethical review and approval was not required for the study on human participants in accordance with the local legislation and institutional requirements. Written informed consent for participation was not required for this study in accordance with the national legislation and the institutional requirements.

## Author Contributions

Conceptualization, HZ, IDP, JBV, and AW. Data curation, IDP and HZ. Formal analysis, IDP and HZ. Funding acquisition, MP, FL, and AW. Investigation, IDP, HZ, and HB. Methodology, IDP and AW. Project administration, IDP, HZ, and AW. Resources, PP, FL, and AW. Supervision, IDP and AW. Visualization, IDP, HZ, and AW. Writing—original draft, IDP, and HZ. Writing—review & editing, HZ, IDP, HB, MP, JBV, FL, and AW. All authors contributed to the article and approved the submitted version.

## Funding

The project was funded by Kom op tegen Kanker (Stand up to Cancer), the Flemish cancer society (OZ7410). IDP and HZ are supported by research grants of Kom op tegen Kanker (Stand up to Cancer), the Flemish cancer society (OZ7410). We would like to thank Mr. Willy Floren for funding some of the equipment used in this study.

## Conflict of Interest

JBV has had in the last three years consulting/advisory relationships with Immunomedics, Innate Pharma, Merck-Serono, Merck Sharp & Dome Corp, PCI Biotech, Synthon Biopharmaceuticals, Debiopharm, Cue Biopharma, and WntResearch and has received honoraria from Merck-Serono, MSD, and BMS.

The remaining authors declare that the research was conducted in the absence of any commercial or financial relationships that could be construed as a potential conflict of interest.

## Publisher’s Note

All claims expressed in this article are solely those of the authors and do not necessarily represent those of their affiliated organizations, or those of the publisher, the editors and the reviewers. Any product that may be evaluated in this article, or claim that may be made by its manufacturer, is not guaranteed or endorsed by the publisher.

## Supplementary Material

Supplementary Figure 1 mRNA expression level of Akt1/2/3 in HNSCC patients, available from TCGA. The graph shows the log transformed mRNA expression (mean and standard deviation) of Akt1/2/3 from 522 HNSCC patients (individual dots). This dataset (TCGA Provisional, RNASeqV2) was downloaded from cBioportal. *, p-value ≤ 0.050.

Supplementary Figure 2 The cytotoxic effect of cetuximab plus MK2206 with a total treatment duration of 72h. **(A)** IC_50_ of MK2206 for HNSCC cell lines after treatment with MK2206 alone and in combination with cetuximab. **(B)** Combination index (CI) versus fraction affected (FA) plot of 2.5 μM MK2206 with fixed doses of cetuximab (25 nM and 50 nM) in SC263-S (red), SCC22b-S (blue), SC263-R (green) and SCC22b-R (orange). *, significant difference in IC_50_ compared to MK2206 monotherapy (p < 0.050). CI < 0.800, CI = 1.000 ± 0.200 and CI > 1.200 indicated synergism, additive effect and antagonism, respectively. Suffix -S: cetuximab sensitive cell line and suffix -R: acquired cetuximab resistant cell line.

Supplementary Figure 3 The cytotoxic effect of cetuximab for 168h with MK2206 added during the last 72h of treatment. **(A)** IC_50_ of MK2206 for cetuximab sensitive HNSCC cell lines after combination treatment. **(B)** IC_50_ of MK2206 for acquired cetuximab sensitive HNSCC cell lines after combination treatment. **(C)** Combination index (CI) versus fraction affected (FA) plot of 1 μM MK2206 with fixed doses of cetuximab (0.5 nM, 1 nM and 5 nM) in SC263-S (red) and SCC22b-S (blue). **(D)** Combination index (CI) versus fraction affected (FA) plot of 1 μM MK2206 with fixed doses of cetuximab (5 nM, 25 nM and 50 nM) in SC263-R (green) and SCC22b-R (orange). *, significant difference in IC_50_ compared to MK2206 monotherapy (p < 0.050). CI < 0.800, CI = 1.000 ± 0.200 and CI > 1.200 indicated synergism, additive effect, and antagonism, respectively. Suffix -S: cetuximab sensitive cell line and suffix -R: acquired cetuximab resistant cell line.
