# Supplementary figures and images for: The Role of Akt in Acquired Cetuximab Resistant Head and Neck Squamous Cell Carcinoma: An In Vitro Study on a Novel Combination Strategy
Source: Front Oncol. 2021 Sep 10;11:697967. doi: 10.3389/fonc.2021.697967 (PMC8462273; doi:10.3389/fonc.2021.697967)

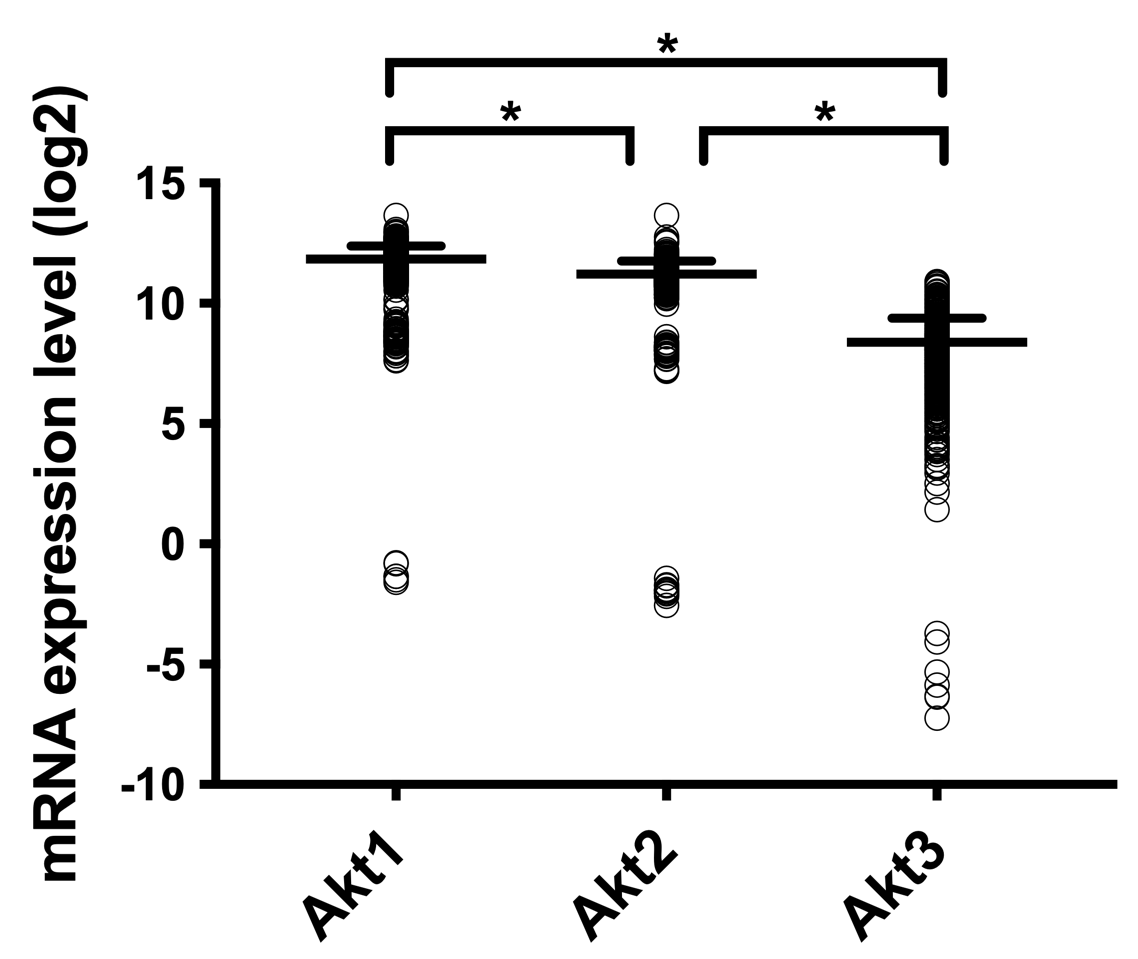

Supplement: Supplementary Figure 1 — mRNA expression level of Akt1/2/3 in HNSCC patients, available from TCGA. The graph shows the log transformed mRNA expression (mean and standard deviation) of Akt1/2/3 from 522 HNSCC patients (individual dots). This dataset (TCGA Provisional, RNASeqV2) was downloaded from cBioportal. *, p-value ≤ 0.050. [file Image_1.tiff]

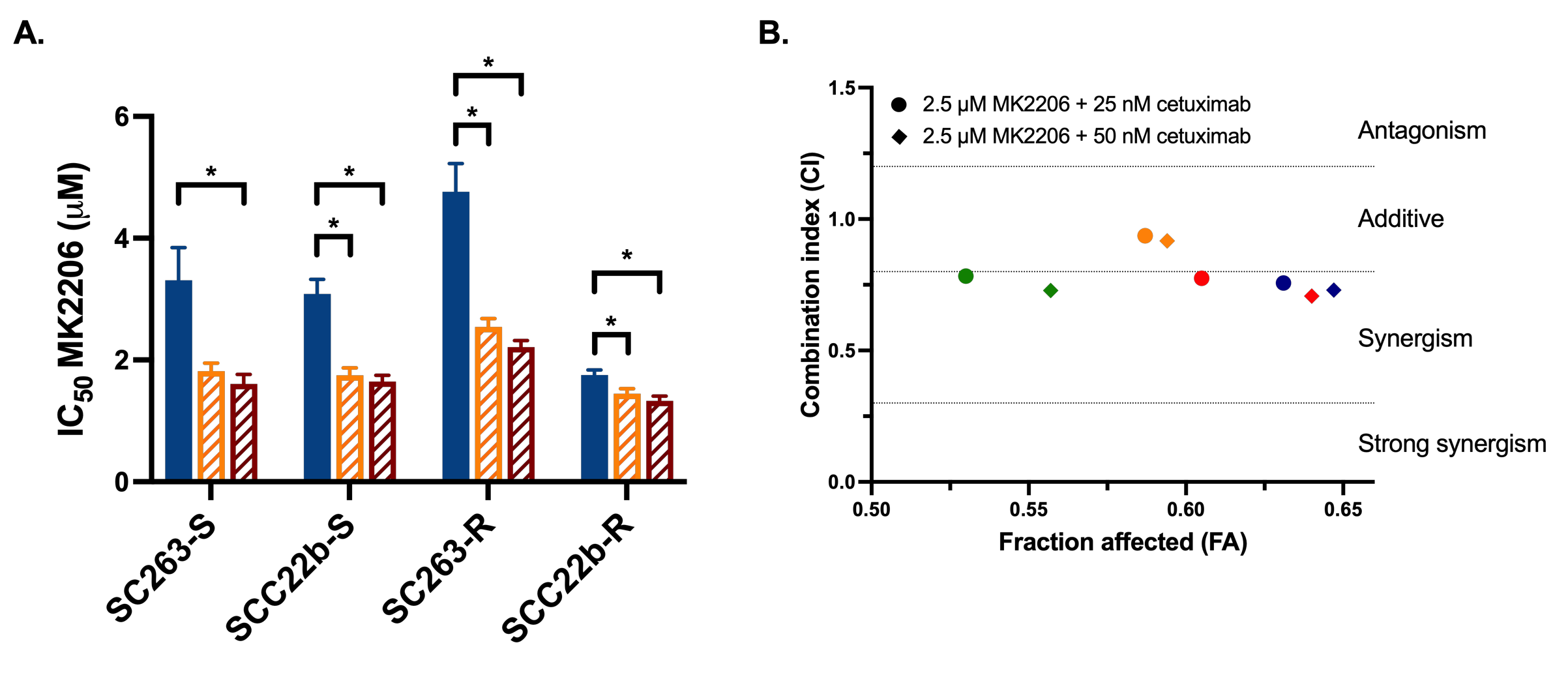

Supplement: Supplementary Figure 2 — The cytotoxic effect of cetuximab plus MK2206 with a total treatment duration of 72h. (A) IC50 of MK2206 for HNSCC cell lines after treatment with MK2206 alone and in combination with cetuximab. (B) Combination index (CI) versus fraction affected (FA) plot of 2.5 μM MK2206 with fixed doses of cetuximab (25 nM and 50 nM) in SC263-S (red), SCC22b-S (blue), SC263-R (green) and SCC22b-R (orange). *, significant difference in IC50 compared to MK2206 monotherapy (p < 0.050). CI < 0.800, CI = 1.000 ± 0.200 and CI > 1.200 indicated synergism, additive effect and antagonism, respectively. Suffix -S: cetuximab sensitive cell line and suffix -R: acquired cetuximab resistant cell line. [file Image_2.tiff]

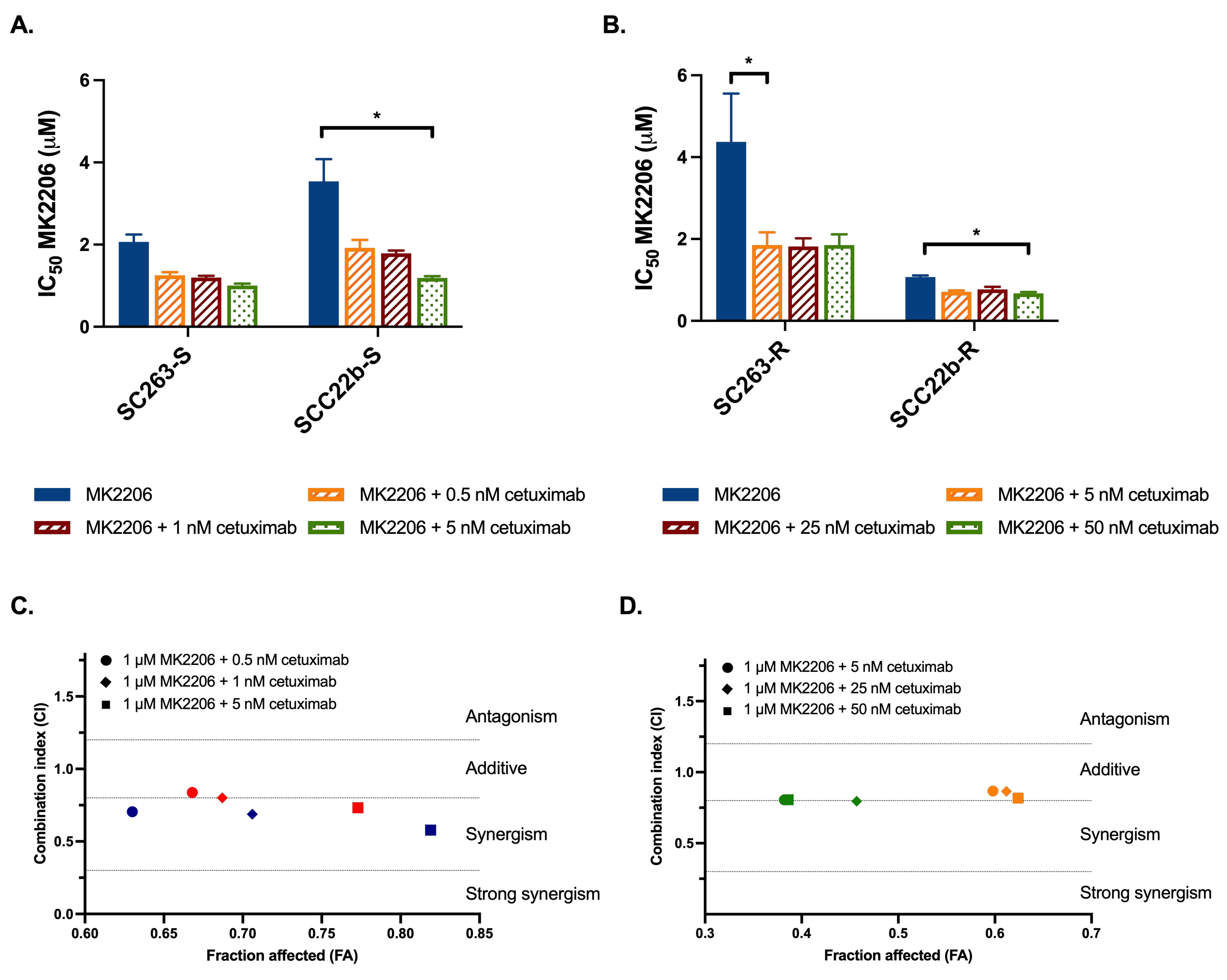

Supplement: Supplementary Figure 3 — The cytotoxic effect of cetuximab for 168h with MK2206 added during the last 72h of treatment. (A) IC50 of MK2206 for cetuximab sensitive HNSCC cell lines after combination treatment. (B) IC50 of MK2206 for acquired cetuximab sensitive HNSCC cell lines after combination treatment. (C) Combination index (CI) versus fraction affected (FA) plot of 1 μM MK2206 with fixed doses of cetuximab (0.5 nM, 1 nM and 5 nM) in SC263-S (red) and SCC22b-S (blue). (D) Combination index (CI) versus fraction affected (FA) plot of 1 μM MK2206 with fixed doses of cetuximab (5 nM, 25 nM and 50 nM) in SC263-R (green) and SCC22b-R (orange). *, significant difference in IC50 compared to MK2206 monotherapy (p < 0.050). CI < 0.800, CI = 1.000 ± 0.200 and CI > 1.200 indicated synergism, additive effect, and antagonism, respectively. Suffix -S: cetuximab sensitive cell line and suffix -R: acquired cetuximab resistant cell line. [file Image_3.tiff]
