# Supplementary material for: The Role of Akt in Acquired Cetuximab Resistant Head and Neck Squamous Cell Carcinoma: An In Vitro Study on a Novel Combination Strategy
Source: Front Oncol. 2021 Sep 10;11:697967. doi: 10.3389/fonc.2021.697967 (PMC8462273; doi:10.3389/fonc.2021.697967)
Supplement: Supplementary file 4 [file Table_1.docx]

Supplementary Material

**Supplementary Table 1.** List of target proteins in the Human Phospho-Kinase Antibody Array (ARY003B, Proteome Profiler, R&D Systems).

| *Target protein* | *Phosphorylation site* |
| --- | --- |
| Akt 1/2/3 (pan) | S473 |
| Akt 1/2/3 (pan) | T308 |
| AMPKα1 | T183 |
| AMPKα2 | T172 |
| β-Catenin | / |
| Chk-2 | T68 |
| c-Jun | S63 |
| CREB | S133 |
| EGFR | Y1086 |
| eNOS | S1177 |
| ERK1/2 | T202/Y204, T185/Y187 |
| FAK | Y397 |
| Fgr | Y412 |
| Fyn | Y420 |
| GSK-3 α/β | S21/S9 |
| Hck | Y411 |
| HSP27 | S78/S82 |
| HSP60 | / |
| JNK 1/2/3 | T183/Y185, T221/Y223 |
| Lck | Y394 |
| Lyn | Y397 |
| MSK1/2 | S376/S360 |
| p27 | T198 |
| p38α | T180/Y182 |
| p53 | S15 |
| p53 | S46 |
| p53 | S392 |
| p70 S6 Kinase | T389 |
| p70 S6 Kinase | T421/S424 |
| PDGF Rβ | Y751 |
| PLC-γ1 | Y783 |
| PRAS40 | T246 |
| Pyk2 | Y402 |
| RSK1/2/3 | S380 |
| Src | Y419 |
| STAT2 | Y689 |
| STAT3 | S727 |
| STAT3 | Y705 |
| STAT5a | Y699 |
| STAT5a/b | Y699 |
| STAT5b | Y699 |
| STAT6 | Y641 |
| TOR | S2448 |
| WNK-1 | T60 |
| Yes | Y426 |

/, total protein levels were determined.
