# Supplementary material for: The Role of Akt in Acquired Cetuximab Resistant Head and Neck Squamous Cell Carcinoma: An In Vitro Study on a Novel Combination Strategy
Source: Front Oncol. 2021 Sep 10;11:697967. doi: 10.3389/fonc.2021.697967 (PMC8462273; doi:10.3389/fonc.2021.697967)
Supplement: Supplementary file 5 [file Table_2.docx]

Supplementary Material

**Supplementary Table 2.** Pairwise comparisons using Tukey-Kramer HSD between mRNA expression of Akt1, Akt2 and Akt3 in HNSCC patients.

| **Comparison** | **Difference in mean** | **Lower 95% confidence interval** | **Upper 95% confidence interval** | **P-value** |
| --- | --- | --- | --- | --- |
| Akt1 versus Akt3 | 3344.401 | 3178.808 | 3509.994 | **<0.0001** |
| Akt2 versus Akt3 | 2064.125 | 1898.532 | 2229.718 | **<0.0001** |
| Akt1 versus Akt2 | 1280.276 | 1114.684 | 1445.869 | **<0.0001** |

P < 0.050, significant difference in mean mRNA expression between Akt isoforms. P < 0.050 are indicated in bold.
